# Malaria Infection Increases Attractiveness of Humans to Mosquitoes

**DOI:** 10.1371/journal.pbio.0030298

**Published:** 2005-08-09

**Authors:** Renaud Lacroix, Wolfgang R Mukabana, Louis Clement Gouagna, Jacob C Koella

**Affiliations:** **1**Laboratoire de Parasitologie Evolutive, Université P. and M. Curie, Paris, France,; **2**Department of Zoology, University of Nairobi, Nairobi, Kenya,; **3**Mbita Point Research and Training Centre, International Centre of Insect Physiology and Ecology, Mbita Point, Kenya; Swiss Tropical InstituteSwitzerland

## Abstract

Do malaria parasites enhance the attractiveness of humans to the parasite's vector? As such manipulation would have important implications for the epidemiology of the disease, the question has been debated for many years. To investigate the issue in a semi-natural situation, we assayed the attractiveness of 12 groups of three western Kenyan children to the main African malaria vector, the mosquito *Anopheles gambiae*. In each group, one child was uninfected, one was naturally infected with the asexual (non-infective) stage of *Plasmodium falciparum,* and one harboured the parasite's gametocytes (the stage transmissible to mosquitoes). The children harbouring gametocytes attracted about twice as many mosquitoes as the two other classes of children. In a second assay of the same children, when the parasites had been cleared with anti-malarial treatment, the attractiveness was similar between the three classes of children. In particular, the children who had previously harboured gametocytes, but had now cleared the parasite, were not more attractive than other children. This ruled out the possibility of a bias due to differential intrinsic attractiveness of the children to mosquitoes and strongly suggests that gametocytes increase the attractiveness of the children.

## Abstract 2

A study of children in Kenya reveals that those individuals harboring the transmissible gametocyte stage of malaria parasites are more attractive to the mosquito vectors that spread this disease.

## Introduction

Malaria remains one of the most important causes of human mortality [1]. This epidemiological success of the parasite is at least partly due to its very high intensity of transmission, leading, on average, to several hundred infections per year in humans living in areas with the most intense transmission [2]. It became clear with the earliest models of the epidemiology of malaria that this intense transmission is largely due to the part of the parasite's life-cycle that takes place within the mosquito vector [3], and in particular to the interaction between the life-span of the mosquito, the duration of the parasite's development, and the mosquito's biting rate. More recently, ideas have moved from the epidemiological description of these interactions to the evolutionary idea that the parasite might manipulate the mosquito's behaviour to enhance its transmission.

Thus, malaria parasites manipulate various aspects of their mosquito vector's biting behaviour in ways that should increase their transmission success [4]. When the parasites have completed their development within the mosquito and have thus developed into the sporozoite stage, which can be transmitted by a bite on a human host, they increase the biting frequency, and thus the rate of contact between mosquitoes and humans and, consequently, the rate of transmission [5]. In contrast, at an earlier developmental stage (i.e., as oocysts, the non-transmissible developmental stage of the parasite), they decrease the biting rate by decreasing the mosquito's motivation to bite [6,7]. Thus, as biting is risky, the probability that the mosquito survives the parasite's development to the transmissible sporozoite stage is increased during early pre-sporozoite development.

During their development within the human, malaria parasites should be expected to manipulate the mosquito's biting behaviour by making infectious humans (i.e., those harbouring gametocytes) more attractive to mosquitoes. This would not only increase the parasite's transmission, but would also have a strong effect on the epidemiology of malaria [8]. However, while increased feeding on infected hosts has been observed in some studies [9,10], others do not show increased attractiveness [11]; the question of behavioural manipulation by gametocytes to increase the host's attractiveness to mosquitoes therefore remains a controversial issue in the biology of malaria.

One of the reasons for the lack of conclusive studies is that individual people vary considerably in their intrinsic attractiveness to mosquitoes [12,13]. Attractiveness depends on, for example, human sweat components [14], body temperature and moisture [15,16], or body odour and breath [13,17]. This background variation makes it difficult to find an effect of manipulation and, more importantly, to be certain that any observed difference is due to infection rather than to a difference in intrinsic attractiveness. Indeed, without a proper control, any observation of increased attractiveness by people infected with gametocytes might mean that harbouring gametocytes is a consequence of being very attractive to mosquitoes (and thus being infected frequently) rather than vice versa. However, the variability of intrinsic attractiveness to mosquitoes could be accounted for with a comparison of two measures of the attractiveness of individuals: the first when they are infected and the second when they have cleared the parasite.

We used this approach in our study, in which we estimated, in semi-natural conditions, the extent to which malaria gametocytes enhance the attractiveness of asymptomatic humans to mosquitoes in the area around Mbita, Nyanza Province, Kenya between April and July 2004 (with the agreement of the Kenya National Ethical Review Committee). We considered 12 groups of three children who were between 3 and 15 y old. This age-class constitutes the main reservoir of gametocytes in the area of Mbita [18]. After we had obtained informed consent by the parents or guardians, apparently healthy children from local primary schools were screened for malaria in the morning. Each day, we chose three children to participate in the study: one who was uninfected, one infected with the asexual (non-infective) stage of the malaria parasite *Plasmodium falciparum,* and one harbouring the parasite's gametocytes (the stage infective to mosquitoes). Note that symptomatic children (i.e., with increased temperature or other symptoms of disease) were immediately referred to the health clinic for proper treatment and therefore did not participate in the study. The attractiveness of the children in each group was measured with a three-way olfactometer consisting of a central chamber attached with PVC tubes to three tents where the three children of the group were resting or sleeping [12] (Figure 1). Mosquitoes were released in the central chamber and given the opportunity to follow their preferred odour. For further details of the set-up, see Materials and Methods.

**Figure 1:**
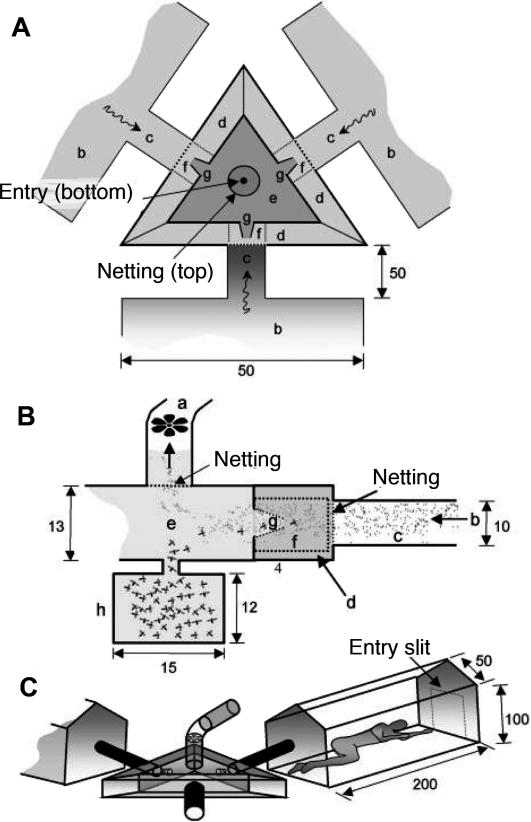
Experimental Set-Up (A)–(C) Top (A), cross-sectional (B), and three-dimensional (C) views of the olfactometer used. The fan (a) draws air (∼130 l/min per tent) from the three tents (b) to the outside environment via PVC pipes (c), trap chambers (d), and a central chamber (e). Each trap chamber contains a collecting cage (f) into which an exit trap opens (g). The fan pipe and release cup (h) are fitted to the top and bottom of the central chamber, respectively. Diagrams are not shown to scale; all dimensions are in centimetres. Source: [12].

To account for the variability of intrinsic attractiveness to mosquitoes, we treated all children with detectable parasitaemia with Fansidar and assayed the same children 2 wk after their first assay. Although it is not clear whether Fansidar kills gametocytes, subsequent microscopic examinations confirmed, in each of the 12 groups, that the children had cleared the parasite before the second assay. Thus, the first assay showed the combined effects of intrinsic and parasite-induced attractiveness, while the second assay gave an indication of the intrinsic variation of attractiveness among the children; the difference between the two assays could be used to analyse the effect of infection above the background variation.

## Results/Discussion

The total number of mosquitoes attracted to the set of children in a group was similar before (20.6 mosquitoes per triplet) and after (18.6 per triplet) treatment with Fansidar (Figure 2A). Before treatment, mosquitoes preferred the gametocyte carriers, so that, on average, 10.2 mosquitoes were attracted by the gametocyte carriers, 5.3 by uninfected children, and 5.4 by children harbouring asexual stages. That the strong attraction of the gametocyte carriers was indeed due to the presence of the gametocytes is suggested by the fact that, after treatment, the children who had previously harboured gametocytes attracted a similar number of mosquitoes (4.4) to both the children who had previously harboured asexual stages (5.8) and the previously uninfected children (8.1). This pattern was confirmed with a statistical analysis that considered the difference between the proportions of the mosquitoes (relative to the 100 or so that were added to the olfactometer) attracted to gametocyte carriers before and after treatment. Indeed, after treatment, mosquitoes were approximately 14% less likely to be attracted to the former gametocyte carriers than before treatment (*t*-test: *t* = 2.23, df = 11, *p* = 0.048; Wilcoxon signed-rank test: *p* = 0.042).

**Figure 2:**
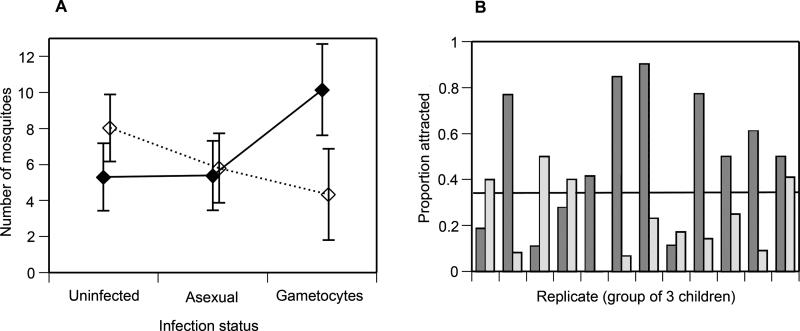
Graphical Representation of Results (A) Number of mosquitoes attracted to each class of children. Points show means of 12 groups; vertical lines show standard errors of the means. Solid diamonds show data of children before treatment; open diamonds denote children after treatment. (B) Proportion of the responsive mosquitoes (i.e., the ones that were attracted to any of the children within a group) attracted to the children who harboured gametocytes (before treatment; dark bars) and to the children who had cleared their gametocytes (after treatment; light bars). The horizontal line shows the proportion expected if the mosquitoes showed no preference.

As a considerable number of mosquitoes did not respond to the odours of the three children (which is often the case when mosquitoes are used in experimental set-ups), we also considered only the mosquitoes that responded to the children's odours and compared the proportion of the mosquitoes within a replicate attracted to the gametocyte carrier before treatment and after treatment (Figure 2B). The figure shows that, in most replicates, the gametocyte carrier attracted more than a third of the mosquitoes before he or she had been treated, but attracted a smaller proportion of the mosquitoes after treatment. This result suggests, again, that the gametocytes enhance the attractiveness of their host. This visual analysis was confirmed by a statistical one, which showed that the difference between the proportion of mosquitoes attracted to the gametocyte carrier before and after the treatment is greater than 0 (df = 11, *p* = 0.039). A Wilcoxon signed-rank test gave a similar result with *p* = 0.052. (Note that the statistically significant finding, despite a small number of groups, emphasises the role of the gametocytes in determining attractiveness to mosquitoes above the intrinsic variation of attractiveness among individuals.)

Our analysis shows that increased attractiveness was not due to an intrinsic attractiveness of gametocyte carriers but to the infection status associated with the presence of gametocytes. The mechanism underlying this manipulation is unknown, but it is likely that the parasites change the infected individual's breath or body odour, as these are involved in attracting mosquitoes at the distances involved in our experiment [15,17]. While transpiration and body temperature also attract mosquitoes at these distances [15], these factors are less likely to be involved in the manipulation as the infection was asymptomatic in all of the children involved in our study.

A striking aspect of our results is that former gametocyte carriers (i.e., after treatment) seem to repel mosquitoes, as only 22%, i.e., less than one third, of the responding mosquitoes prefer these children (*t*-test: *t* = −2.203, df = 11, *p* = 0.050; Wilcoxon signed-rank test: *p* = 0.040). An explanation for this result could be based on the slight anaemia in previously infected children. Mosquitoes might sense this anaemia and prefer those children with a higher concentration of red blood cells as it is these that the mosquitoes require. This would indicate a remarkable adaptation by the mosquitoes. The interpretation, however, would predict that the children previously infected with the asexual stage should also repel mosquitoes—but this was not observed. Indeed, as 47% of the mosquitoes preferred these children, there was a tendency for the opposite effect (*t*-test: *t* = 1.65, df = 11, *p* = 0.127; Wilcoxon signed-rank test: *p* = 0.233).

In conclusion, our data suggest that mosquitoes are more attracted to humans infected by the transmissible gametocyte stage of malaria parasites than to uninfected individuals or individuals infected with asexual, non-transmissible stages. Previous studies have shown that malaria also manipulates the mosquito's biting behaviour at the oocyst stage (when infection decreases the motivation to bite and thereby increases the probability that the mosquito survives the parasite's development) [6,7] and at the sporozoite stage (when infection increases both the motivation to bite and the biting frequency) [5,7]. Thus this study completes the picture by demonstrating that the gametocyte, the only transmission stage from the human to the mosquito, manipulates the biting behaviour of its vector to enhance its transmission to the vector.

Apart from the diversity of mechanisms employed by the parasite to manipulate its vector and increase its transmission, what is striking about the set of demonstrations of behavioural manipulation is that the parasite appears to influence the parameters that are most critical for transmission and thus for the parasite's fitness [3]: the biting rate of the mosquitoes when they become infected and when they infect humans and, consequently, the mosquito's mortality during the parasite's development. Such manipulation therefore has a profound effect on the epidemiology of disease and, if it is not considered, can lead to severe biases in our estimates of the intensity of malaria transmission.

## Materials and Methods

Screening for malaria was carried out with thick blood smears, stained with 10% Giemsa and examined microscopically with (100x) oil-immersion lens for the presence of sexual and asexual parasites. As we did not use molecular techniques, infections with low densities of parasites may have been missed. Thus our results compare, strictly speaking, individuals with no parasites or a low level of parasitaemia with individuals with more intense infections, rather than comparing uninfected with infected individuals. In addition, microscopy and Giemsa staining detect only those gametocytes circulating in the blood, so that we missed the sequestered gametocytes. However, as mosquitoes do not pick up sequestered gametocytes, missing these parasites in an assay is indeed desired.

Attractiveness of the children in each group to mosquitoes was measured with a three-way olfactometer consisting of a central chamber attached to three tents [12] (see Figure 1). At sunset, the volunteer children entered the tents to rest or sleep. The infection status of the children was changed randomly among tents among the 12 replicates, so that any residual odour in the olfactometer could not bias the results (but rather, at most, increase random variation). The tents were connected with PVC tubes to a central cage, where a fan was placed to draw air from the tents. About 100 mosquitoes were released into the central cage, and were given 30 min to follow their preferred odour into the entry traps placed between the PVC tubes and tents. The mosquitoes were uninfected females from a colony of *Anopheles gambiae* s.s. that had been established from specimens collected in Njage village, Kilobero District, Tanzania in 1996 and is maintained at the ICIPE laboratory. We used females 5 d after emergence that had had no prior access to blood, had been maintained on glucose solution (6%) provided with a cotton wick, and had been starved (i.e., provided with water only) for 6 h before the experiment.
